# Is Demographic Concordance Between Lifestyle Coach and Participant Associated With Participant Retention and Weight Change in the National Diabetes Prevention Program Lifestyle Change Intervention?

**DOI:** 10.1155/jdr/7663175

**Published:** 2026-03-26

**Authors:** Britney P. Smart, Anna Forte, Jacquelyn Jacobs, Janna Simon, Rebeca Gordenstein, Elise Ramos

**Affiliations:** ^1^ Sinai Urban Health Institute, Sinai Chicago, Chicago, Illinois, USA; ^2^ Department of Community Health Sciences, School of Public Health, University of Illinois Chicago, Chicago, Illinois, USA, uic.edu; ^3^ Illinois Public Health Institute, Chicago, Illinois, USA

**Keywords:** demographic concordance, diabetes, National Diabetes Prevention Program lifestyle change program, program retention, weight loss

## Abstract

**Objective:**

The goal of this study is to explore the association between racial/ethnic, sex, and age category concordance and participant retention and weight loss among participants enrolled in the National Diabetes Prevention Program lifestyle change program (National DPP LCP).

**Methods:**

This analysis was conducted using a sample of 653 participants who were enrolled in a 12‐month National DPP LCP at one of 17 Chicago community‐based organizations between June 2019 and July 2023. Using multilevel logistic regression modeling, we examined the association between participant lifestyle–coach racial/ethnic, sex, and age category concordance and retention, as well as weight loss. All models controlled for coach education, coach training, program‐engagement level, as well as participant education, sex, age, and race/ethnicity if not already accounted for in the concordance variable.

**Results:**

In adjusted models, we observed a statistically significant relationship between race/ethnic concordance and increased odds of retention. There was no significant relationship between demographic concordance and weight change > = 3*%*.

**Conclusions:**

Our findings suggest that there is increased odds of retention among racially concordant pairs. We did not observe a significant relationship between gender and age concordant pairs and retention nor race, gender, and age concordant pairs and weight loss. Our findings suggest that National DPP LCP programs could consider racial concordance in their program design and implementation as a factor to boost participant retention.

## 1. Introduction

Diabetes impacts more than 37 million Americans and is the eighth leading cause of death in the United States as of 2021 [1]. Among those living with diabetes, 90%–95% of those are diagnosed with Type 2 diabetes [2]. Furthermore, since 2019 approximately 96 million US adults live with prediabetes—a condition in which blood glucose levels are higher than normal, but not high enough to be a clinical diagnosis of diabetes [1]. There are also well‐documented and pervasive disparities in rates of Type 2 diabetes in the United States by race, ethnicity, sex, and age. According to the Center for Disease Control and Prevention′s (CDC) National Diabetes Statistics report, 17.4% of Black adults and 15.0% of Hispanic adults have been diagnosed with diabetes, whereas the prevalence among non‐Hispanic white adults is approximately 13.6% [2]. Further, diabetes prevalence is highest among adults older than 65 years (29.2%) and slightly higher among men (15.4%) when compared with women (14.1%) [1]. With steady increases in diabetes prevalence and the disproportionate impact on communities of color, men, and older adults, it is critical to identify effective solutions and implementation strategies aimed at preventing Type 2 diabetes. One evidence‐based intervention to support people living with prediabetes is the National Diabetes Prevention Program lifestyle change program (National DPP LCP).

The National DPP LCP is a structured, evidence‐based intervention aimed at preventing or delaying the onset of Type 2 diabetes among adults with prediabetes or at high risk of developing Type 2 diabetes [3]. The National DPP LCP uses a standardized 1‐year curriculum, facilitated by trained lifestyle coaches, to help participants build healthy habits focused on nutrition and physical activity [4–6]. In a participant level evaluation using registry data for a cohort of 35,844 adults, researchers have found that, on average, participants achieved a 4.2% decrease in their weight, and 41.8% of participants met their activity goal of 150 min per week [7]. The literature also highlights the relationship between participant retention and the program′s efficacy, with participants experiencing a 0.3% reduction in weight for every additional session attended [7]. In a 10‐year follow‐up of participants enrolled in a National DPP LCP, one study found that the lifestyle changes prompted by participation in the program were associated with delayed onset of diabetes for approximately 4 years [6]. Given this established link between participant attendance and weight loss, understanding facilitators of participant attendance will provide critical actionable guidance to improve participant outcomes. Previous studies have shown strong relationships between program effectiveness and factors such as class delivery mode (virtual vs. in‐person), behavioral changes (food logging vs. physical activity), and the level of coach–client interaction, but little is known about the impact of demographic concordance on retention and successful participation in the program [8–11].

Many studies in recent years have examined the relationship between demographic concordance and improved patient outcomes in a variety of health care settings [12–17]. Although research has shown positive associations with patient–provider concordance and patient satisfaction, quality, and a reduction in health care expenditures, the findings are inconsistent across all demographic factors and even less is known about the impact of concordance on clinical outcomes [12, 13, 15, 18]. A 2020 study found decreased mortality rates among Black neonates when being cared for by a Black physician, but did not observe any association between maternal mortality and mother–physician racial concordance [19]. In regard to biological sex concordance, Greenwood et al. found that among female patients, being in a sex‐discordant physician–patient relationship was associated with higher mortality rates for heart attack patients; however, there was no association for male patients [20]. Although the existing literature on the association between demographic concordance and health outcomes is mixed, the findings are strongest among patients from marginalized backgrounds, implying that concordance can be an important facilitator for reducing disparities in clinical outcomes [19–22]. Considering the rampant disparities in rates of diabetes in the United States, further research is needed to determine whether demographic concordance can result in improved participant retention, ultimately improving weight loss among participants in a National DPP LCP. The objective of this paper is to test the relationship between racial/ethnic, sex, and age category concordance and participant retention and weight loss among participants enrolled in an approved National DPP LCP in the city of Chicago.

## 2. Materials and Methods

In this paper we use multilevel logistic regression modeling to determine the extent to which participant lifestyle coach demographic concordance can influence a measurable change in two outcomes for the target population: retention and weight change. To address multiple facets of program implementation, the broader evaluation used the RE‐AIM framework (reach, effectiveness, adoption, implementation, and maintenance) as a guide [23]. This analysis focuses on participant retention and weight change as two measures of effectiveness. The Mount Sinai Hospital Institutional Review Board deemed this retrospective analysis exempt.

### 2.1. Sample

Between 2019 and 2023, 17 Chicago organizations launched a total of 84 National DPP LCPs. The sample for this paper comes from the 52 programs that provided at least 6 months of data by the end of the data collection period in July 2023. Participants were recruited into a National DPP LCP after attending a recruitment session (discovery session/Session 0), a community health fair, or learning about the program from a community organization, local health clinic, or social media. Individuals were eligible if they were 18 years of age or older, had been told by a clinician they have prediabetes or had an A1c between 5.7% and 6.4% in the last year, had a previous diagnosis of gestational diabetes, or scored at least 5 on the American Diabetes Association′s Prediabetes Risk Screener [24]. Individuals previously diagnosed with Type 1 or Type 2 diabetes or who were pregnant at the time of enrollment were ineligible to participate in the program.

### 2.2. Data Collection

Data for this analysis came from Diabetes Prevention Recognition Program (DPRP) datasets, lifestyle coach profiles, quarterly progress reports, and internal fidelity checklists.

#### 2.2.1. DPRP Datasets

Each organization must submit biannual DPRP datasets to the CDC to achieve full recognition. For the present study, the evaluation team requested quarterly submissions in the same format as required by the CDC. National DPP LCP session data were collected and entered by lifestyle coaches into each program′s preferred database (i.e., Redcap, Excel, DAPS). The following variables were only collected at enrollment: sociodemographic variables (sex at birth, gender identity, age, race, ethnicity and education), enrollment motivation, enrollment source, payer source, participant′s state, and prediabetes determination (i.e., glucose test, A1c, diagnosis of gestational diabetes, or a risk test). The following variables were collected at each session: *s*ession type (i.e., core session, core maintenance session, ongoing maintenance session, or makeup session), date of session, weight, and physical activity minutes. The 2021 DPRP Data Guidance contains a full list of DPRP variables [25]. The evaluation team collated all submitted excel data into one file for analysis.

#### 2.2.2. Lifestyle Coach Profiles

Lifestyle coach profiles were collected from each lifestyle coach upon completion of their lifestyle coach training or upon program initiation (for previously trained coaches). Profile sheets included site and cohort identifiers, as well as the following coach sociodemographic variables: sex, race, ethnicity, age, and education. The evaluation team entered all data into an Excel file upon receipt and appended it to the DPRP dataset for analysis.

#### 2.2.3. Quarterly Progress Reports

Quarterly reports were required grant deliverables for each organization. Reports focused on progress and milestones for the respective reporting period and included details on additional support strategies and wraparound services designed to address barriers to participant enrollment and retention. More specifically, quarterly reports allowed the evaluation team to categorize programs as low‐touch and high‐touch.

#### 2.2.4. Internal Fidelity Checklists

Fidelity checklist packets were developed by the evaluation team and included session‐specific checklists, a program enhancement log and a referral form. Session‐specific checklists and the enhancement log allowed coaches to indicate which parts of the session were completed, and note deviations, incentives, and program enhancements. The referral form was used to document external referrals to external activities, programs and/or services outside of the class. Fidelity checklists allowed the evaluation team to link coaches to their respective cohorts and extract information to inform the program engagement level variable.

### 2.3. Variables

#### 2.3.1. Outcome Variables

Retention: A binary retention variable was created using two National DPP measures: the number of attended sessions per person and total number of sessions for the particular program that someone was in. Total number of sessions was collected via the National DPP data collection sheet. Based on the CDC′s evaluation guidance, retention is defined as having attended eight sessions in 6 months. Retention was dichotomized due to programmatic definitions as well as to align with the current literature [26].

Weight loss: A binary weight loss variable was created using two DPRP measures: participant weight and date of session. The most recent reported weight was subtracted from the first reported weight to calculate weight change (measured in both pounds and percent change). These variables were used to create a new binary indicator “weight loss”, where observations were coded “1” where weight loss was greater than 3% and “0” where weight loss was less than 3%. The 3% threshold was used based on the CDC′s evaluation guidance. Weight loss was dichotomized due to programmatic definitions as well as to align with the current literature [27].

#### 2.3.2. Independent Variables

Three binary concordance variables (age category, race/ethnicity, and sex) were created to indicate where participant and lifestyle coach demographics matched. These variables were coded “1” where demographic attributes matched and “0” where they did not. Participant demographic data were collected and coded as a series of binary variables (consistent with CDC requirements), with one variable for each: Hispanic, non‐Hispanic Black, non‐Hispanic White, American Indian or Alaskan Native, Asian, Native Hawaiian or other Pacific Islander. These data were transformed into a single race/ethnicity variable and then compared with coach race/ethnicity data to determine matches.

#### 2.3.3. Covariates

Adjusted model covariates include coach education, coach training (related to behavioral interventions), program engagement level (high‐touch or low‐touch, based on level of coach engagement with the participant outside of class, and program supports offered), and participant demographics (education, sex, age, and race/ethnicity). Coach education is a categorical variable with five groups: Grade 12/GED, some college, college graduate, some graduate level work, and masters or more. Coach training is a continuous variable that describes the total number of trainings attended. Program engagement level is a binary variable that indicates whether the program was a high‐touch program (coded “1”) or a low‐touch program (coded “0”). Participant education was a categorical variable (18–24 years, 25–34 years, 35–44 years, 45–54 years, 55 years or older, and not reported), as was participant education (less than Grade 12, Grade 12 or GED, some college or technical school, college or technical school graduate or higher, and not reported). Participant race and ethnicity variables were generated from self‐reported binary variables for Hispanic/Latine (yes/no), American Indian or Alaska Native (yes/no), Asian (yes/no), Black (yes/no), native Hawaiian or other Pacific Islander (yes/no), and White (yes/no). These data were used to create a single new race/ethnicity covariate with three categories: Black, White, and Hispanic/Latine; participant racial categories which made up less than 2% of the sample were dropped from the analysis.

### 2.4. Statistical Analysis

Univariate and bivariate analyses were used to describe our sample and describe the distribution of our key predictors by the outcomes of interest. Multilevel logistic regression was used to model the relationship between concordance variables and categorical outcome measures, accounting for nesting by the organizational and cohort level. The research team used analytical methods described in Sommet and Morselli and Leyland et al. following a three‐step approach to building a multilevel model; first running a null model to examine clustering at different levels, then building our intermediate model to determine variation between clusters, and finally running our final model [28, 29]. In our sample, there were 653 participants, with clustering at both the organizational (*K* = 11) and cohort level (*K* = 29). We found an intraclass correlation coefficient of 0.05 for the organization and 0.27 for the cohort level, meaning that 5% of the observed variance was explained by between‐organization differences and 28% of the observed variation was explained by between‐cohort differences. Although we did not observe meaningful deviance in our intermediate model, we proceeded with the final multilevel model due to guidance provided in Sommet and Morselli. As a sensitivity analysis, we also conducted a binary logistic regression to assess the robustness of findings; results can be found in the supporting information (Tables S1 and S2). Models were adjusted for coach education, coach training, program engagement level, as well as participant education, sex, age, and race/ethnicity. In models where participant demographic factors were already accounted for via the concordance variable, we did not include that variable as a covariate. For example, in our model examining the relationship between racial/ethnic concordance and retention, we did not include participant race/ethnicity as a covariate. For our primary models, those examining the relationship between demographic concordance and retention, we also calculated risk differences to aid in the interpretation of our findings. All analyses were conducted using Stata for Windows, Version 15.1 [30].

## 3. Results

Data for 653 participants were included in this analysis. On average, participants in the sample were female (84.9%), 55 years or older (36.7%), and identified their race/ethnicity as Hispanic/Latine (54.8%) (Table 1). In total, there were 22 lifestyle coaches who led cohorts, the majority of whom were female (81.8%), identified as Hispanic/Latine (54.5%), were 25–34 years old (42.8%), and had more than a college education (90.9%) (Table 2). We saw the largest proportion of participant/coach concordance for sex (73.8%) and the lowest concordance for participant/coach age (19.4%).

**Table 1:** Participant descriptive data.

Participant demographics (654)	N/M	%/SD
Sex		
Male	99	15.01
Female	555	84.99
Age category		
18–24 years	17	2.60
25–34 years	75	11.49
35–44 years	156	23.89
45–54 years	165	25.27
55+ years	240	36.75
Race/ethnicity		
Black	263	40.28
White	32	4.90
Hispanic/Latine	358	54.82
Education		
Less than Grade 12	112	17.15
Grade 12 or GED	97	14.85
Some college/tech	117	17.92
College/tech	113	17.3
Not reported	214	32.77
Completed eight sessions in 6 months	461	70.60
Engagement level		
Low touch	353	54.06
High touch	300	45.94
Concordance		
Racial/ethnic	435	66.62
Age category	127	19.45
Sex	482	73.81
Weight Change (lbs)	5.548	11.94
Weight percentage change decreased > = 3	237	36.29

**Table 2:** Lifestyle coach descriptive data.

Coach demographics (*N* = 22)	Number (*N*)/mean (M)	Percent (%)/standard deviation (SD)
Sex		
Male	4	18.18
Female	18	81.82
Race/ethnicity		
Hispanic	12	54.55
White	4	18.18
Black	5	22.73
Asian	1	4.55
Age		
18–24	1	4.76
25–34	9	42.86
35–44	2	9.52
45–54	6	28.57
55+	3	14.29
Education		
Grade 12 or GED	1	4.55
Some college	1	4.55
College graduate	9	40.91
Some graduate level work	2	9.09
Masters or more	9	40.91
Total training	4.54	2.78

### 3.1. Retention

**Figure 1:**
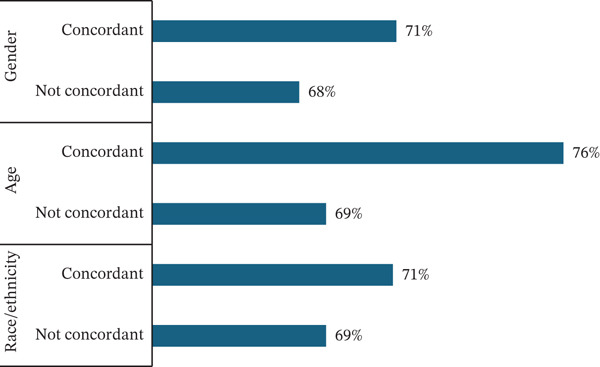
Proportion retained by demographic concordance.

**Figure 2:**
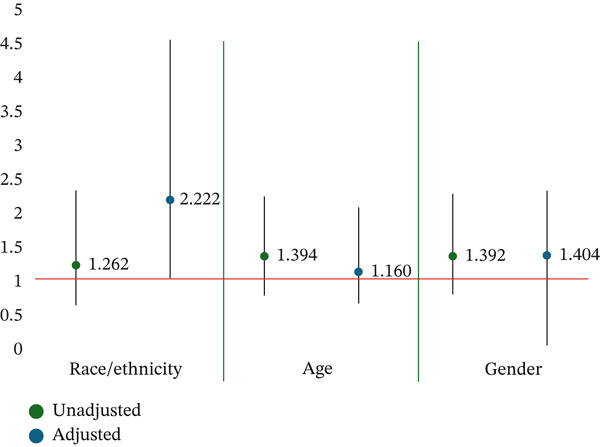
Odds of participant retention as a function of demographic concordance.

Approximately 70.6% of the participants enrolled in the program were retained, completing eight sessions within 6 months. Of those participants, 71% were in a race/ethnic concordant dyad, 76% were in an age concordant dyad, and 71% were in a sex concordant dyad (Figure 1). Among participants who were not retained, although not statistically significant, overall rates of concordance were lower across different demographic characteristics, 28% for race/ethnicity, 23% for age, and 28% for sex (Table 3). In unadjusted models, when compared with participants where there was a mismatch between participant and coach race/ethnicity (OR: 1.26, *p* value: 0.46), age (OR: 1.39, *p* value: 0.21), and sex (OR: 1.392, *p* value: 0.198), congruent participant/coach dyads had increased odds of being retained in the program (Table 4; Figure 2). In adjusted models, we saw similar patterns, with race/ethnicity (OR: 2.22, *p* value: 0.03), age (OR: 1.16, *p* value: 0.48), and sex concordance (OR: 1.40, *p* value: 0.19) associated with increased odds of retention; however, the relationship was only statistically significant for race/ethnicity. We also calculated risk difference for our primary models of interest, finding that gender concordance was associated with a 5% change in absolute risk (CI: −0.02, 0.12), racial concordance was associated with an 11% absolute change in risk (CI: 0.01, 0.21), and for age concordance there was a 3% change in absolute risk (CI: −0.05, 0.10). Additionally, when comparing the fit of our mixed effects model to a logistic regression model, we saw improved fit for our model′s examining retention and racial/ethnically concordant pairs and our gender concordant pairs.

**Table 3:** Participant demographics by retention status.

	Not retained	Retained	
	*N*(%)/M(SD)	*N*(%)/M(SD)	*p*value
Sex			
Male	37 (37.8)	61 (62.2)	0.049
Female	155 (27.9)	400 (72.1)	
Age Category			
18–24 years	8 (47.1)	9 (52.9)	0.009
25–34 years	25 (33.3)	50 (66.7)	
35–44 years	35 (22.4)	121 (77.6)	
45–54 years	62 (37.6)	103 (62.4)	
55+ years	62 (25.8)	178 (74.2)	
Race/Ethnicity			
Black	85 (32.3)	178 (67.7)	0.271
White	11 (34.4)	21(65.6)	
Hispanic/Latine	96 (26.8)	262 (73.2)	
Education			
Less than Grade 12	21 (18.8)	91 (81.3)	< 0.001
Grade 12 or GED	30 (30.9)	67 (69.1)	
Some college/tech	30 (25.6)	87 (74.4)	
College/tech	22 (19.5)	91 (80.5)	
Not reported	89 (41.6)	125 (59.4)	
Engagement Level			
Low touch	115 (32.6)	238 (67.4)	0.053
High touch	77 (25.7)	223 (74.3)	
Concordance			
Racial/ethnic			
Concordant	125(28.7)	310(71.3)	0.597
Not concordant	67 (30.7)	151 (69.3)	
Age category			
Concordant	30 (23.6)	97 (76.4)	0.111
Not concordant	162 (30.8)	364 (69.2)	
Gender			
Concordant	138 (28.6)	344 (71.4)	0.467
Not concordant	54 (31.6)	117 (68.4)	
Weight change (lbs)	1.28(6.0)	7.21 (13.3)	0.000
Weight decreased > = 3*%*	16 (6.8)	221 (93.2)	< 0.001

**Table 4:** Multilevel Logistic Regression modeling retention as a function of demographic concordance.

	Unadjusted				Adjusted			
	Odds Ratio (OR)	*p*value	LCI	HCI	OR	*p*value	LCI	HCI
Race/Ethnicity Concordance	1.26	0.465	0.67	2.35	2.22	0.030	1.078	4.577
Age Category Concordance	1.39	0.219	0.82	2.36	1.16	0.484	0.702	2.106
Sex Concordance	1.39	0.198	0.84	2.30	1.40	0.196	0.838	2.352

^∗^Adjusted for coach education, coach training, engagement level, participant race/ethnicity, age, sex, and education.

### 3.2. Weight Change

**Figure 3:**
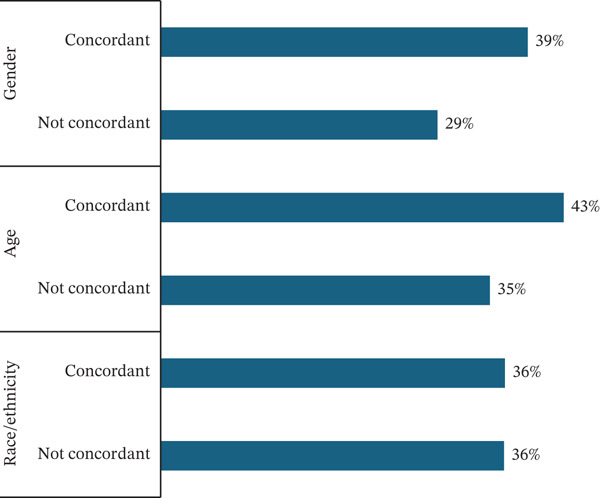
The proportion of participants who achieved > = 3% change in weight and demographic concordance.

**Figure 4:**
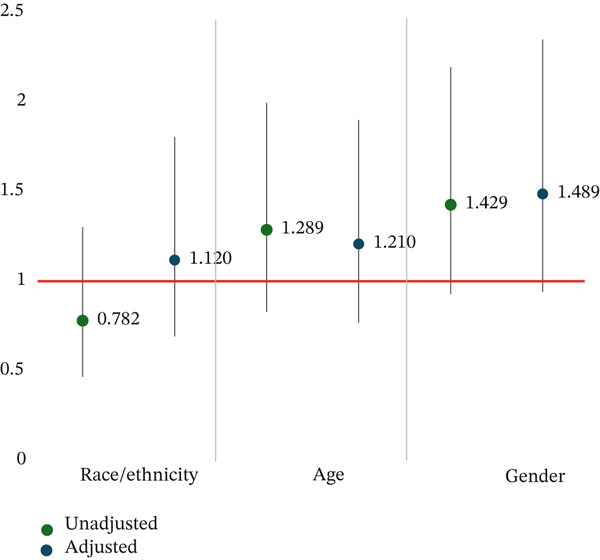
Odds of participant weight loss > = 3% as a function of demographic concordance.

In our sample, 36% of participants experienced a ≥ 3% decrease in weight by their last session in the program. Among participants who experienced a ≥ 3% decrease in their weight, 36% experienced concordance by race/ethnicity, 43% experienced age category concordance, and 39% experienced sex concordance (Figure 3). Among participants whose weight decrease was < 3%, 63% experienced race/ethnicity concordance, 57% experienced age category concordance, and 61% experienced sex concordance (Table 5). In unadjusted and adjusted models, we saw no significant relationship between demographic concordance and weight change ≥ 3*%* (Table 6, Figure 4). We observed improved fit when comparing our mixed effects models with a logistic regression in our models exploring the relationship between weight change and racially/ethnically and gender concordant pairs.

**Table 5:** Participant demographics by percent weight change.

	*W* *e* *i* *g* *h* *t* *D* *e* *c* *r* *e* *a* *s* *e* < 3*%*	*W* *e* *i* *g* *h* *t* *D* *e* *c* *r* *e* *a* *s* *e* *d* > = 3*%*	
	*N*(%)	*N*(%)	*p*value
Sex			
Male	69 (70.4)	29 (29.6)	0.134
Female	347 (62.5)	208 (37.5)	
Age Category			
18–24 years	13 (76.4)	4 (25.5)	0.320
25–34 years	49 (65.3)	26 (34.67)	
35–44 years	92 (59.0)	64 (41.0)	
45–54 years	113 (68.5)	52 (31.5)	
55+ years	149 (62.1)	91 (37.9)	
Race/Ethnicity			
Black	181 (68.8)	82 (31.2)	0.030
White	23 (71.9)	9 (28.1)	
Hispanic/Latine	212 (59.2)	146 (40.8)	
Education			
Less than Grade 12	60 (53.6)	52 (46.4)	0.015
Grade 12 or GED	60 (61.9)	37 (38.1)	
Some College/tech	75 (64.1)	42 (35.9)	
College/tech	67 (59.3)	46 (40.7)	
Not reported	154 (72.0)	60 (28.0)	
Engagement Level			
Low touch	238 (67.4)	115 (32.6)	0.032
High touch	178 (59.3)	122 (40.7)	
Concordance			
Racial/ethnic	277 (63.7)	158 (36.3)	0.983
Age Category	73 (57.5)	54 (42.5)	0.104
Sex	295 (61.2)	187 (38.8)	0.026
Retained	240(52.1)	221 (47.9)	0.000

**Table 6:** Multilevel logistic regression modeling percent weight change as a function of demographic concordance.

	Unadjusted				Adjusted			
	OR	*p*value	LCI	HCI	OR	*p*value	LCI	HCI
Race/ethnicity concordance	0.78	0.347	0.469	1.304	1.12	0.641	0.695	1.806
Age category concordance	1.29	0.254	0.832	1.997	1.21	0.407	0.770	1.901
Sex concordance	1.43	0.103	0.930	2.196	1.49	0.087	0.943	2.350

*Note:* Adjusted for coach education, coach training, engagement level, race/ethnicity, age, sex, education.

## 4. Discussion

In the present study, we used demographic data from participants and lifestyle coaches, and program data from the implementation of a 5‐year expansion of the National DPP LCP in Chicago to examine the association between participant–coach demographic concordance on retention and weight loss. To our knowledge, this is the first study to examine these relationships among patients with prediabetes participating in a lifestyle change program to prevent the onset of diabetes. Our findings suggest that participant–coach racial/ethnic concordance may be a significant predictor of retention in the program, as measured by attending at least eight sessions in the first 6 months of the year‐long program. Although age category and sex concordance were also associated with an increase in retention, neither of those demographic factors was statistically significant in the adjusted or unadjusted models.

There is evidence from existing literature that patient–provider demographic concordance may influence patient satisfaction, medication adherence, healthcare utilization, and engagement in research [12, 17, 27]. However, no studies have measured the association between participant–coach concordance within the context of preventive health interventions. Further, our study examined the association between participant–coach concordance and two outcomes (participant retention and weight loss). The effectiveness of many lifestyle change interventions, including the National DPP LCP, is associated with attendance in the program; therefore, it is critical to build evidence for implementation strategies that will contribute to program adherence [8, 9].

Racial/ethnic concordance in the participant–coach relationship is one implementation strategy that has strong potential to improve participant outcomes. It is hypothesized that this concordance may lead to more effective communication and trust building. Jetty et al. found that racial concordance led to lower emergency department utilization and lower healthcare expenditures [12]. Similarly, our study found that racial/ethnic concordance led to a significantly higher rate of retention compared with nonconcordant dyads. We hypothesize that having the same race or ethnicity as one′s lifestyle change coach leads to perceived similarities in lived experience and ultimately greater trust and retention in the program.

Although we found racial/ethnic concordance to be a significant predictor of participant retention, we did not find this to be the case for sex and age category concordance or weight loss. We had fewer age congruent dyads, which is reflective of the older participant population, and on average, younger lifestyle coaches. It is also possible we did not find statistically significant differences because of how the specific age categories were constructed. Individuals whose age was close to a cutoff point may be assigned to different age groups despite a small absolute age difference. Yao et al. examined the relationship between age and sex concordance as it relates to adherence to statin medication and also failed to find a statistically significant association [17]. Weight loss is a subsequent outcome associated with the intervention and therefore the factors that contribute to weight loss are substantially more complex than facilitator demographics. These factors include genetic factors, other comorbidities, and initial weight loss success [31].

In the present study, we calculated the odds of retention to explore varying strength of participant–coach concordance as an implementation strategy for an evidence‐based program that, when implemented with fidelity, has the potential to delay onset of diabetes. It is possible that we did not find a statistically significant relationship between retention and sex and age category concordance because the measured outcomes were not sensitive to these factors. These demographic factors may instead drive outcomes like participant satisfaction with the program and delivery of the intervention, or even clinical outcomes.

This study has several strengths. First, many studies have examined outcomes associated with patient–provider concordance, but this is the first to focus on facilitator–participant concordance within the context of a public health intervention. Second, most of the literature on patient–provider concordance includes a higher proportion of non‐Hispanic White dyads than any other racial or ethnic group. This oversampling is likely due to the fact that physicians of color are substantially underrepresented in the physician workforce [32]. However, our study is unique in that 81% of lifestyle coaches were non‐White, which allows for more meaningful conclusions around the effects of racial/ethnic concordance. Finally, the current study has a large sample size of more than 650 adults who participated in this lifestyle change program, which contributes to a potentially smaller margin of error in our analysis.

There are also limitations that must be noted. First, the demographic categories were created based on the CDC′s reporting and evaluation guidance and therefore may not be representative of one′s specific ethnic identity (e.g., there is no distinction between Hispanic origin groups such as Mexican, Puerto Rican, etc.). Similarly, the age cutoffs were based on the CDC reporting structure. We were also limited to biological sex because gender identity was not collected for the entirety of the evaluation. As described above, individuals whose age were around the cutoff point may be close in age but placed into separate age categories for analysis. Second, demographic data was all self‐reported by either the participate or the lifestyle coach. Our hypothesis is driven by the theory that an individual’s retention may be influenced by their perceived connection to a facilitator, however our measurement was based on facilitator self‐report, not participant perception of the facilitator’s demographic characterization. Finally, we did not explore the association between coach prediabetes disease status and retention, which may be an important predictor of retention as similar lived experience as it relates to disease management, may foster a stronger relationship between participants and coaches.

## 5. Conclusion

Decisions about staffing lifestyle change interventions, which seek to change complex behaviors and address health outcomes, must be an intentional process that centers the needs of program participants. In addition to ensuring that program facilitators are well‐trained, exhibit strong facilitation skills, and are knowledgeable about the context, facilitator demographic features might be considered, particularly race and ethnicity which can foster trust and comfort for matched participants. It is therefore important to consider a workforce that mirrors the population served. Further, organizations that implement lifestyle change programs should ensure adequate opportunities for professional development that will strengthen existing staff and create a pipeline for underrepresented minorities in the healthcare workforce. Future research should explore the causal pathway between participant–facilitator concordance and retention. Specifically, factors through which racial concordance acts on retention such as trust, similarities in lived experience, or even diabetes disease status. There is also an opportunity to examine how age, race/ethnicity, and sex concordance influence retention through qualitative or mixed methods approaches.

## Funding

This study was supported by the Centers for Disease Control and Prevention (10.13039/100000030) (NU58DP006625‐01‐00).

## Disclosure

The contents are those of the authors and do not necessarily represent the official views of, nor an endorsement, by CDC or the US Government.

## Ethics Statement

This project was supported by the Centers for Disease Control and Prevention (CDC), under cooperative agreement Number 1 NU58DP006625‐01‐00. This evaluation and analysis were reviewed by the Mount Sinai Hospital Institutional Review Board and were deemed to be exempt.

## Conflicts of Interest

The authors declare no conflicts of interest.

## Supporting information


**Supporting Information** Additional supporting information can be found online in the Supporting Information section. Tables S1 and S2: Binary logistic regression models testing the relationship between demographic concordance variables and retention and weight change > = 3*%*.

## Data Availability

The data that support the findings of this study are available on request from the corresponding author. The data are not publicly available due to privacy or ethical restrictions.
