# Supplementary material for: Is Demographic Concordance Between Lifestyle Coach and Participant Associated With Participant Retention and Weight Change in the National Diabetes Prevention Program Lifestyle Change Intervention?
Source: J Diabetes Res. 2026 Mar 26;2026:7663175. doi: 10.1155/jdr/7663175 (PMC13140893; doi:10.1155/jdr/7663175)
Supplement: Supplementary file 1 — Supporting Information Additional supporting information can be found online in the Supporting Information section. Tables S1 and S2: Binary logistic regression models testing the relationship between demographic concordance variables and retention and weight change > = 3%. [file JDR-2026-7663175-s001.docx]

| Table S1. Binary Logistic Regression modeling retention as a function of demographic concordance | | | | | | | | |  |
| --- | --- | --- | --- | --- | --- | --- | --- | --- | --- |
|  | **Unadjusted** | | | | **Adjusted** | | | |  |
|  | **OR** | **P value** | **LCI** | **HCI** | **OR** | **P value** | **LCI** | **HCI** |  |
| **Race/Ethnicity Concordance** | 1.208 | 0.263 | 0.867 | 1.685 | 2.43 | 0.011 | 1.222 | 4.86 |  |
| **Age Concordance** | 1.393 | 0.123 | 0.913 | 2.125 | 1.393 | 0.256 | 0.785 | 2.472 |  |
| **Gender Concordance** | 1.147 | 0.446 | 0.805 | 1.636 | 1.468 | 0.251 | 0.761 | 2.833 |  |
| *Adjusted for coach education, coach training, engagement level, Race/ethnicity, age, gender, education, and organization | | | | | | | | |  |
|  |  |  |  |  |  |  |  |  |  |

| Table S2. Binary Logistic Regression modeling weight change as a function of demographic concordance | | | | | | | | |  |
| --- | --- | --- | --- | --- | --- | --- | --- | --- | --- |
|  | **Unadjusted** | | | | **Adjusted** | | | |  |
|  | **OR** | **P value** | **LCI** | **HCI** | **OR** | **P value** | **LCI** | **HCI** |  |
| **Race/Ethnicity Concordance** | 1.00 | 0.983 | 0.715 | 1.407 | 0.88 | 0.702 | 0.454 | 1.699 |  |
| **Age Concordance** | 1.39 | 0.105 | 0.934 | 2.057 | 1.25 | 0.387 | 0.752 | 2.08 |  |
| **Gender Concordance** | 1.53 | 0.026 | 1.052 | 2.236 | 1.64 | 0.134 | 0.858 | 3.148 |  |
| *Adjusted for coach education, coach training, engagement level, Race/ethnicity, age, gender, education, and organization | | | | | | | | |  |
|  |  |  |  |  |  |  |  |  |  |
